# Supplementary material for: A splicing isoform of TEAD4 attenuates the Hippo–YAP signalling to inhibit tumour proliferation
Source: Nat Commun. 2016 Jun 13;7:ncomms11840. doi: 10.1038/ncomms11840 (PMC4909989; doi:10.1038/ncomms11840)
Supplement: Supplementary Information — Supplementary Figures 1-5 and Table 1. [file ncomms11840-s1.pdf]

Supplemental Material for

A splicing isoform of TEAD4 attenuates Hippo-YAP signaling to inhibit tumor proliferation

Yangfan Qi, Jing Yu, Wei Han, Xiaojuan Fan, Haili Qian, Huanhuan Wei, Yi-hsuan S. Tsai, Jinyao Zhao, Wenjing Zhang, Quentin Liu, Songshu Meng, Yang Wang and Zefeng Wang

Supplementary Figure 1 to 5 and legends

Supplementary Table 1

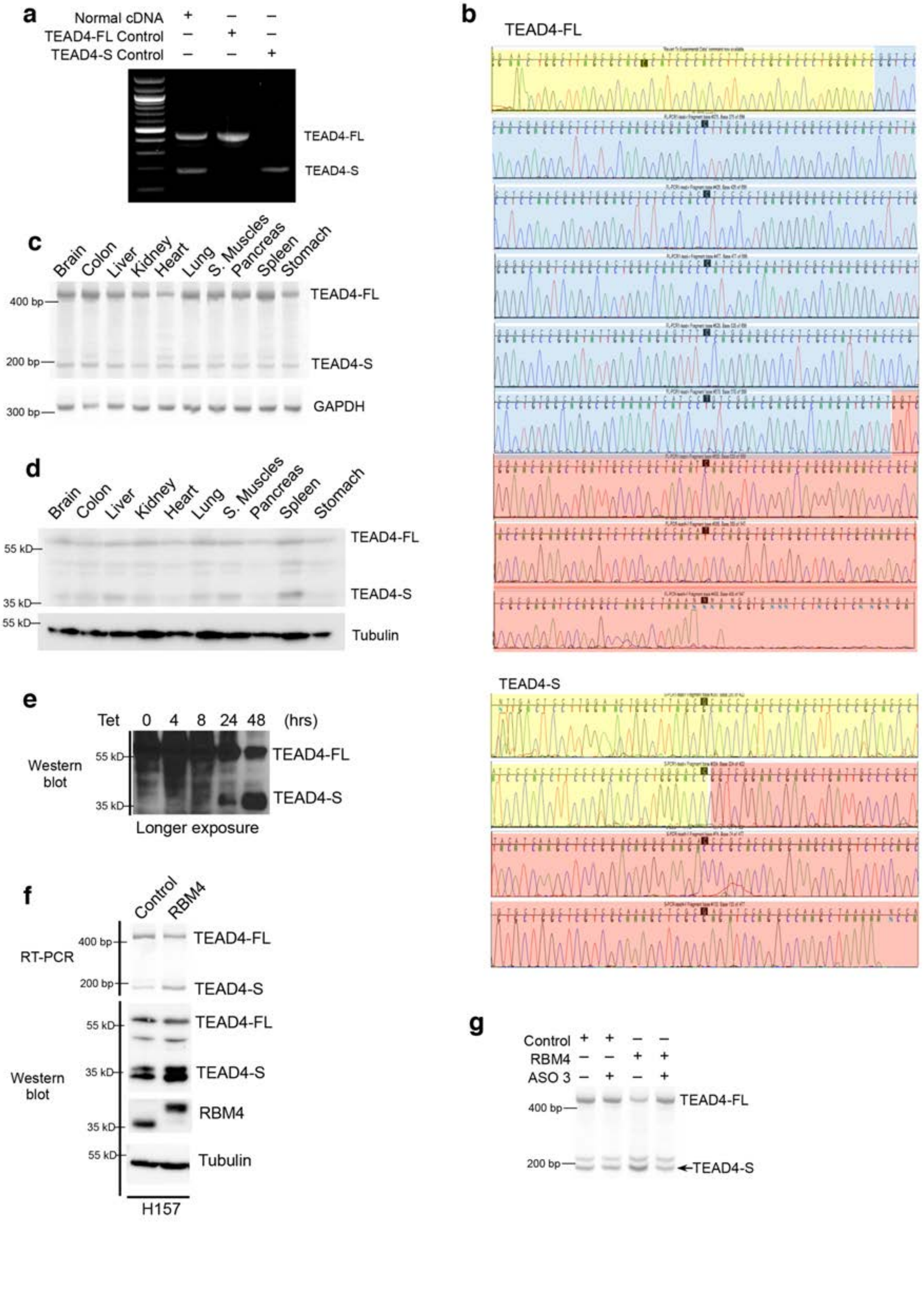

a
Normal cDNA + − −
TEAD4-FL Control − + −
TEAD4-S Control − − +
TEAD4-FL
TEAD4-S
b
TEAD4-FL
TEAD4-S
c
Brain Colon Liver Kidney Heart Lung S. Muscles Pancreas Spleen Stomach
400 bp
200 bp
300 bp
TEAD4-FL
TEAD4-S
GAPDH
d
Brain Colon Liver Kidney Heart Lung S. Muscles Pancreas Spleen Stomach
55 kD
35 kD
55 kD
TEAD4-FL
TEAD4-S
Tubulin
e
Tet 0 4 8 24 48 (hrs)
Western blot
55 kD
35 kD
TEAD4-FL
TEAD4-S
Longer exposure
f
Control RBM4
RT-PCR
400 bp
200 bp
TEAD4-FL
TEAD4-S
Western blot
55 kD
35 kD
35 kD
55 kD
TEAD4-FL
TEAD4-S
RBM4
Tubulin
H157
g
Control + + − −
RBM4 − − + +
ASO 3 − + − +
400 bp
200 bp
TEAD4-FL
TEAD4-S

Supplementary Figure 1. A novel splicing isoform of TEAD4 is regulated by RBM4. (**a**) Tead4 primers were designed to examine both TEAD4-FL and TEAD4-S products simultaneously. cDNAs from H157 cells were used as templates to amplify the two isoforms. The upper and lower products (lane 1) was cut from the gel and purified. The purified PCR products were used as templates to do a second PCR. The resulting PCR products (lane 2 and 3) were purified and sequenced. **(b**) The chromatograph of the sequenced TEAD4 PCR products. The yellow sequences indicate part of exon 2 of TEAD4, the blue sequences indicate the entire exon 3 of TEAD4, and the red sequences indicate part of exon 4 of TEAD4. **(c-d)** The expression levels of two TEAD4 splicing isoforms in different human tissues. A panel of human tissue cDNAs were used as templates to amplify TEAD4 isoforms, and the PCR products were run on a 10% TBE-PAGE gel to separate. The representative gel figure was shown in (c). A panel of human tissue proteins were applied for a western blot assay. The two isoforms of TEAD4 were blotted with anti-TEAD4 antibody as shown in (d). **(e)** 293 cells expressing RBM4 upon tetracycline induction were collected at different time points after induction to determine the expression levels of TEAD4-FL and TEAD4-S (longer exposure). **(f)** RBM4 regulates the splicing of TEAD4 in H157 cells. The cells were stably transfected with RBM4 or vector control and the splicing of TEAD4 was examined by RT-PCR. The representative gels were shown. (**g**) The antisense oligo that masks RBM4 binding site was co-transfected with the RBM4 expression vector or control vector into A549 cells, and the splicing of TEAD4 was determined by RT-PCR.

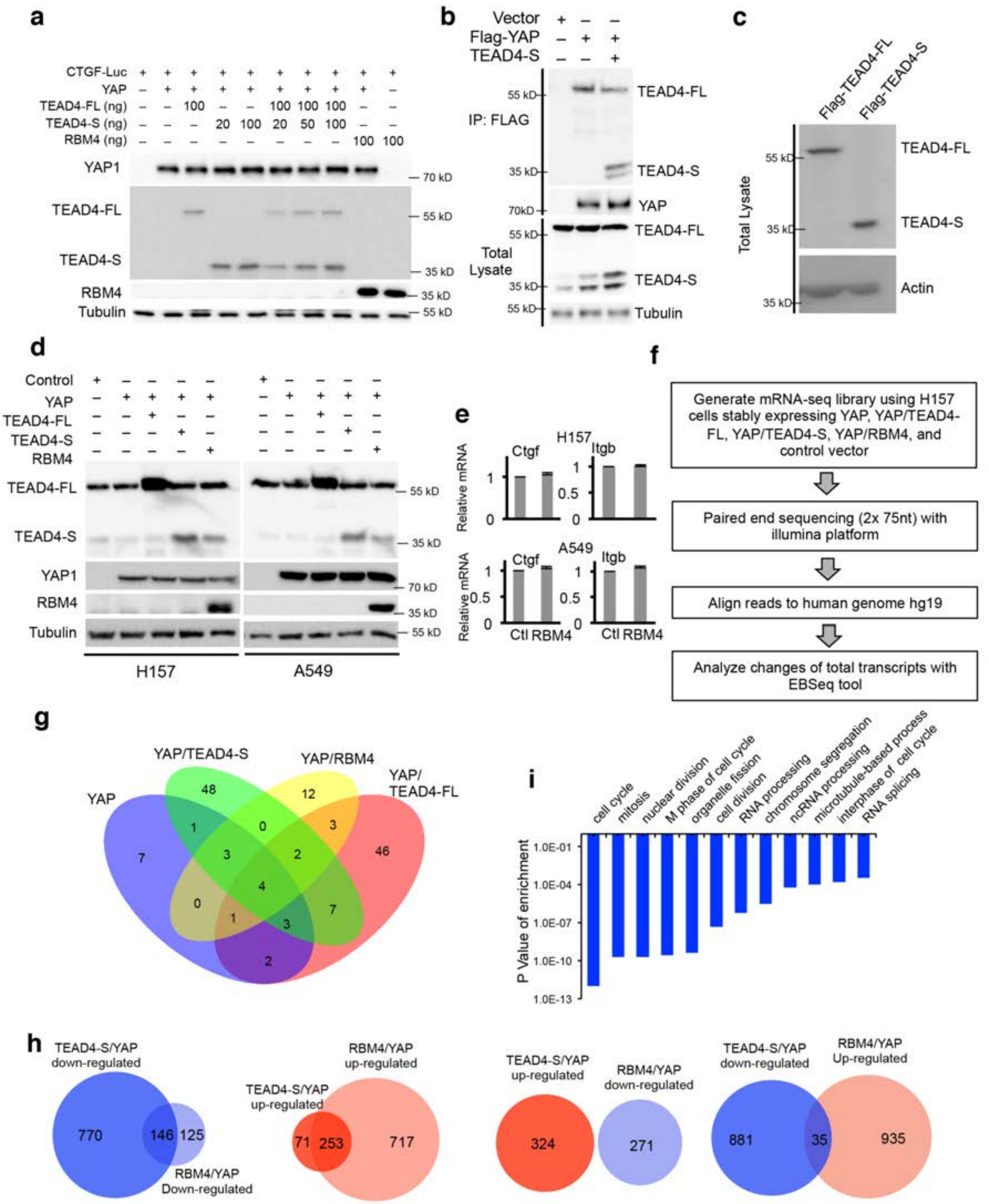

Supplementary Figure 2. TEAD4-S isoform antagonizes full length TEAD4, repressing YAP signaling. (a) TEAD4-FL, TEAD-S, or RBM4 were co-transfected with CTGF luciferase reporter in the presence or absence of YAP into 293T cells. The expression levels of these proteins were determined by western blot with anti-TEAD4,

anti-YAP, anti-RBM4 antibodies. **(b)** 293T cells were co-transfected with control, Flag-YAP and /or untagged TEAD4-S vectors. Proteins were extracted from the transfected cells after 72 hours later. The purified proteins were used for immunoprecipitation assay with anti-Flag antibody. The interaction between Flag-YAP and endogenous TEAD4-FL was examined. The expression of TEAD4-FL and TEAD4-S were determined with anti-TEAD4 antibody using the total lysate. **(c)** The aliquots of the same set of samples for ChIP (in figure 2d) were used for a western blot to examine the expression levels of TEAD4. **(d)** Proteins from the same set of samples for realtime RT-PCR (in figure 2e-2f) were used for a western blot to examine the expression of TEAD4, YAP1, and RBM4 with anti-TEAD4, anti-YAP, and anti-RBM4 antibodies. **(e)** The expression levels of CTGF and ITGB were examined by realtime RT-PCR in RBM4 overexpressed or control H157 and A549 cells. The mean +/- SD of relative mRNA levels from triplicate experiments were plotted. **(f)** The flow chart of the RNA-seq experiment. **(g)** The overlappings between significantly affected genes from the RNA-seq data of cells with co-expression of YAP with TEAD4-FL or TEAD4-S were shown. The data suggested that cells with co-expression of YAP with TEAD4-FL or TEAD4-S displayed limited overlapping between significantly affected genes. **(h)** The overlappings of genes up-regulated or down-regulated upon YAP/TEAD4-S expression with those altered in YAP/RBM4 in RNA-seq data were analyzed. They are significantly overlapped. ($P<2.2\times10^{-16}$) **(i)** The gene ontology analysis of the overlapped genes from the RNA-seq data.

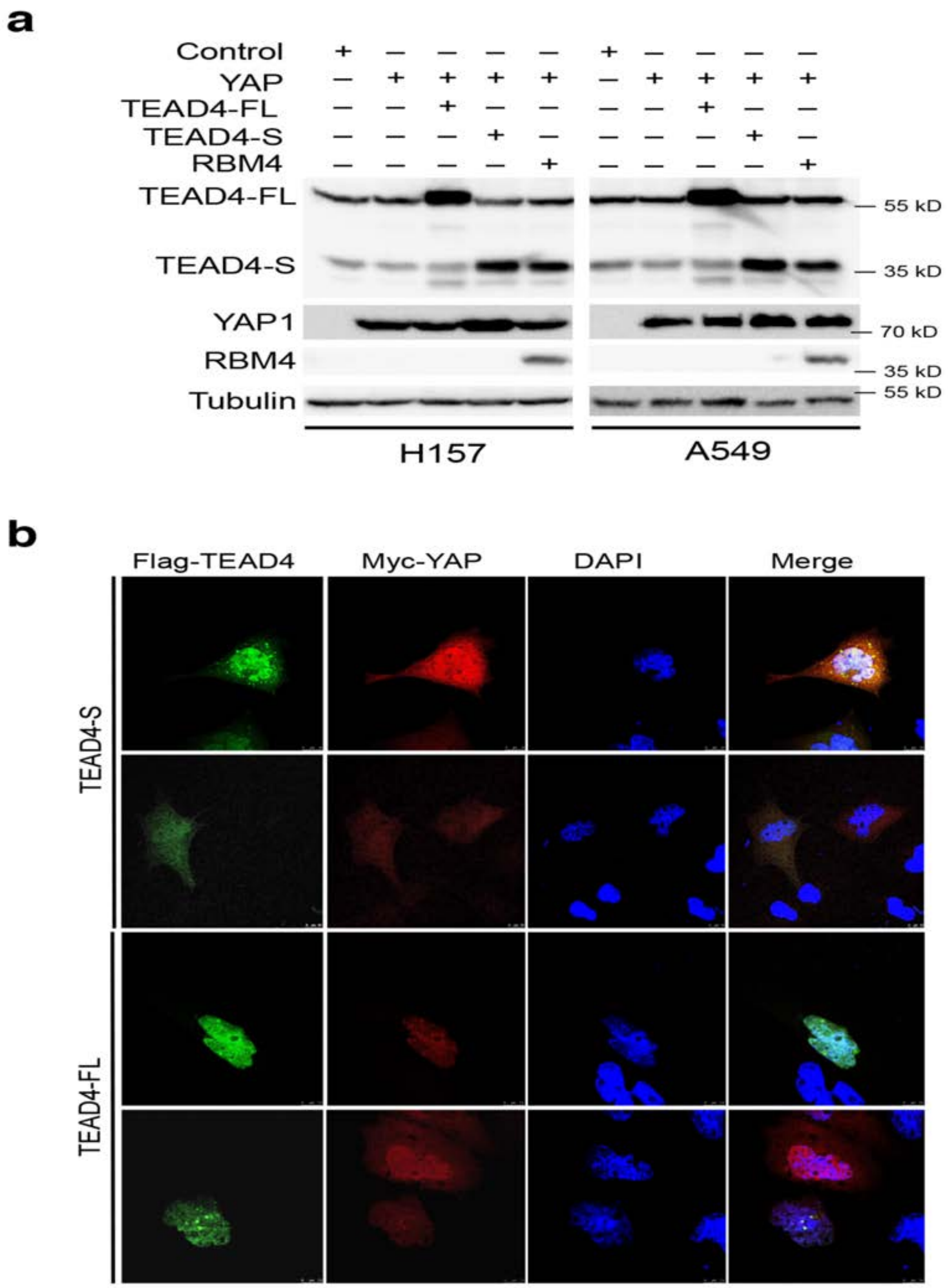

Supplementary Figure 3. (a) H157 and A549 cells that stably expressing YAP alone or co-expressing YAP with TEAD4-FL, TEAD4-S or RBM4 were collected. The protein expression levels of YAP, TEAD4-FL, TEAD4-S, and RBM4 were determined with western blot using anti TEAD4, YAP1, and RBM4 antibodies. **(b)** The cells were co-

transfected with myc-YAP and Flag-tagged TEAD4-FL or TEAD4-S. Then the cells were stained with anti-flag and anti-myc antibodies and the localizations of YAP (red) and TEAD4 (green) were observed under an immunofluorescence microscope.

a

Cancer types	No. of Normal	No. of Tumor
BLCA	23	214
BRCA	110	991
COAD	27	423
HNSC	46	428
KIRC	76	509
KIRP	34	164
LIHC	54	150
LUAD	61	477
PRAD	49	259
THCA	62	495
UCEC	32	491

b

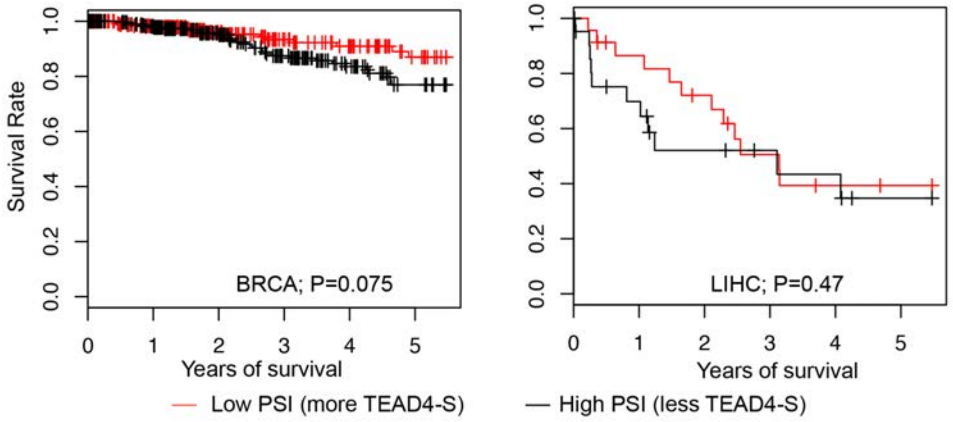

Supplementary Figure 4. TEAD4-S is positively correlated to cancer patients' survival. (a) The cancer types, numbers of the normal and tumor tissues of the TCGA dataset obtained for analyzing the survival were shown. **(b)** The correlations of TEAD4-S with breast cancer and liver cancer patient survivals were demonstrated.

```
TEAD2_Human      MGEPRAGAALDDGSGWTGSEEG-SEEGTGGSEGAGGDGGPDAEGVWSPDIEQSFQEALAI
TEAD3_Human      ----------MASNSWNASSSP-GEAREDGPEGLDKGLDNDAEGVWSPDIEQSFQEALAI
TEAD1_Human      ----------MEPSSWSGSESP-AENMERMSDSADKPIDNDAEGVWSPDIEQSFQEALAI
TEAD4_Human      ---MEGTAGTITSNEWSSPTSPEGSTASGGSQALDKPIDNDAEGVWSPDIEQSFQEALAI
                              . *... .  ..     .:. .   . ********************

TEAD2_Human      YPPCGRRKIILSDEGKMYGRNELIARYIKLRTGKTRTRKQVSSHIQVLARRKSREIQSKL
TEAD3_Human      YPPCGRRKIILSDEGKMYGRNELIARYIKLRTGKTRTRKQVSSHIQVLARKKVREYQVGI
TEAD1_Human      YPPCGRRKIILSDEGKMYGRNELIARYIKLRTGKTRTRKQVSSHIQVLARRKSRDFHSKL
TEAD4_Human      YPPCGRRKIILSDEGKMYGRNELIARYIKLRTGKTRTRKQVSSHIQVLARRKAREIQAKL
                 **************************************************.* *: :  :

TEAD2_Human      K----DQVSKDKAFQTMATMSSAQLISAPSLQAKLGPT----GPQASELFQFWSGGS--G
TEAD3_Human      KAMNLDQVSKDKALQSMASMSSAQIVSASVLQNKFSPPSPLPQAVFSTSSRFWSSPPLLG
TEAD1_Human      K----DQTAKDKALQHMAAMSSAQIVSATAIHNKLGLPG-IPRPTFPGAPGFWPGMIQTG
TEAD4_Human      K----DQAAKDKALQSMAAMSSAQIISATAFHSSMALA---RGPGRPAVSGFWQGALP-G
                 *    **.:****:* **:*****::**. :: .:. .     .  .    ** .    *

TEAD2_Human      -PPWNVPDVKPFSQTPFTLSLTPPSTDLPGYEPPQALSPLPP-PTPSPPAWQARGLGTAR
TEAD3_Human      QQPGPSQDIKPFAQPAYPIQ-PPLPPTLSSYEPLA---PLPS-AAASVPVWQDRTIASSR
TEAD1_Human      -QPGSSQDVKPFVQQAYPIQ-PAVTAPIPGFEPASA--PAPS-----VPAWQGRSIGTTK
TEAD4_Human      -QAGTSHDVKPFSQQTYAVQ-PPL--PLPGFESPAG--PAPSPSAPPAPPWQGRSVASSK
                   .    *:*** * .:.:. ..    :..:*.     * *.      * ** * :.::.

TEAD2_Human      LQLVEFSAFVEPPDAVDSYQRHLFVHISQHCPSPGAPPLESVDVRQIYDKFPEKKGGLRE
TEAD3_Human      LRLLEYSAFMEVQRDPDTYSKHLFVHIGQTNPAFSDPPLEAVDVRQIYDKFPEKKGGLKE
TEAD1_Human      LRLVEFSAFLEQQRDPDSYNKHLFVHIGHANHSYSDPLLESVDIRQIYDKFPEKKGGLKE
TEAD4_Human      LWMLEFSAFLEQQQDPDTYNKHLFVHIGQSSPSYSDPYLEAVDIRQIYDKFPEKKGGLKD
                 *.::*:***:*     *:*..******.:   :  .  * **:**:**************.:

TEAD2_Human      LYDRGPPHAFFLVKFWADLNWGPSGEEAGAGGSISSGGFYGVSSQYESLEHMTLTCSSKV
TEAD3_Human      LYEKGPPNAFFLVKFWADLNSTIQEG---------PGAFYGVSSQYSSADSMTISVSTKV
TEAD1_Human      LFGKGPQNAFFLVKFWADLNCNIQDD---------AGAFYGVTSQYESSENMTVTCSTKV
TEAD4_Human      LFERGPSNAFFLVKFWADLNTNIEDE---------GSSFYGVSSQYESPENMIITCSTKV
                 *: .** :************   .            ..****:***.* : * :: *:**

TEAD2_Human      CSFGKQVVEKVETERAQLEDGRFVYRLLRSPMCEYLVNFLHKLRQLPERYMMNSVLENFT
TEAD3_Human      CSFGKQVVEKVETEYARLENGRFVYRIHRSPMCEYMINFIHKLKHLPEKYMMNSVLENFT
TEAD1_Human      CSFGKQVVEKVETEYARFENGRFVYRINRSPMCEYMINFIHKLKHLPEKYMMNSVLENFT
TEAD4_Human      CSFGKQVVEKVETEYARYENGHYSYRIHRSPLCEYMINFIHKLKHLPEKYMMNSVLENFT
                 ************** *. *:*.: **: ***:***::**:***.:***.***********

TEAD2_Human      ILQVVTNRDTQELLLCTAYVFEVSTSERGAQHHIYRLVRD
TEAD3_Human      ILQVVTSRDSQETLLVIAFVFEVSTSEHGAQHHVYKLVKD
TEAD1_Human      ILLVVTNRDTQETLLCMACVFEVSNSEHGAQHHIYRLVKD
TEAD4_Human      ILQVVTNRDTQETLLCIAYVFEVSASEHGAQHHIYRLVKE
                 ** ***.**:** **  * ***** **.*****:*.**.:
```

Supplementary Figure 5. Multiple sequence alignment of TEAD1 to TEAD4. All TEAD paralogs can generate N-terminal truncated isoform through alternative splicing (TEAD2, TEAD3 and TEAD4) or alternative promoter (TEAD1). The conserved DNA binding motif is labeled by blue bar under the aligned sequences, and the regions that are absent in truncated isoforms are highlighted in yellow. The sequences were aligned with ClustalW2.

Supplementary Table 1. Lists of oligonucleotide used in this study

Primer #	Name	Sequence	Notes
1	TEAD4-F	GGACTCCTTGGAACTGGCTTA	The primer pair used for testing TEAD4
2	TEAD4-R	TTTAGCTTGGCCTGGATCTC	
3	Tead4 Exon 2 fwd	cac GCTAGC AACGATCGCCGCGGCCGGAA	Primers used for cloning TEAD4 exon 2 to 4 into pCDNA3 vector
4	Tead4 intron 2 rev	AAATTAGCCAGGTGTGGGGGCCTCAAGTGCCT	
5	Tead4 intron 2-3 fwd	AGGCACTTGAGGCCCC CACACCTGGCTAATTT	
6	Tead4 intron 3 rev	GGGCATGGGGGGTGCATGCCTTGCAGAGCAGG	
7	Tead4 intron 3-4 fwd	CCTGCTCTGCAAGGCATGCACCCCCCATGCCC	
8	Tead4 exon 4 rev	CAC GCGGCCGC CTGCTTCCTGGTGCGGGTCT	
9	TEAD4-mut1-f	GGAGCCTTGGAGGGCACttataCACCATTACCTCCAAC	The primer pair used for mutating the RBM4 binding site in TEAD4 reporter
10	TEAD4-mut1-r	GTTGGAGGTAATGGTGtataaGTGCCCTCCAAGGCTCC	
11	TEAD4-mut2-f	GGAGCCTTGGAGGGCAgtaacgCACCATTACCTCCAAC	The primer pair used for rescuing the RBM4 binding site with another motif in TEAD4 reporter
12	TEAD4-mut2-r	GTTGGAGGTAATGGTGcgttacTGCCCTCCAAGGCTCC	
13	TEAD4 RIP test fwd	CCATTACCTCCAACGAGTGG	The primer pair used for testing TEAD4 in RIP assay
14	TEAD4 RIP test rev	CTCGTCCGACAGGATGATTT	
15	CTGF-fwd	CCGTACTCCCAAAATCTCCA	The primer pair used for testing CTGF with RT-PCR
16	CTGF-rev	GTAATGGCAGGCACAGGTCT	
17	ITGB2-fwd	TTCTGCAGCAATGGAGTGAC	The primer pair used for testing ITGB2 with RT-PCR
18	ITGB2-rev	ACTGCTCCTGGATGCACTCT	
19	Pum-F1	CACGGATCCTCCCCCCCAAGAAAAAGAGG AAGGTATCTAGAGGCCGCAGCCGCCTTTTG	Encodes NLS between BamHI and XbaI sites
20	Pum-R1	GTGGTCGACTTACCCTAAGTCAACACC	Encodes a stop codon and SalI site
